# Gordon McVie: his legacy as a blueprint for cancer advocates as we strive towards the 2030 global health and sustainable development goals

**DOI:** 10.3332/ecancer.2022.1341

**Published:** 2022-01-13

**Authors:** Gabriella Pravettoni, Marianna Masiero, Christine Mugo-Sitati, Julie Torode

**Affiliations:** 1 Department of Oncology and Hemato-Oncology, University of Milan, Milan 20122, Italy; 2 Applied Research Division for Cognitive and Psychological Science, IEO European Institute of Oncology, IRCCS, Milan 20141, Italy; 3 Kenyan Network of Cancer Organizations, PO Box 106383-00101, Nairobi, Kenya; 4 Institute of Cancer Policy, Global Oncology Group, King’s College London SE1 9RT, UK

**Keywords:** advocacy, cancer control, implementation research, equity, patient-centred, empowerment, decision-making, technologies, health systems resilience, sustainable development goals

## Abstract

Cancer prevention and control services worldwide must actively rebuild and contribute to improved health systems resilience alongside and beyond the COVID-19 (SARS-CoV-2 coronavirus disease) pandemic, especially in low- and middle-income countries. Cancer advocacy groups should respond to this unprecedented challenge as an opportunity to bolster community and patient involvement in research and clinical practice that is adjusted to local needs and circumstances. This short communication provides a synthesis of these critical challenges and, stemming from the pioneering activities of Gordon McVie on patient empowerment, urges policy makers and researchers to develop new implementation strategies that start from the social, economic and health consequences of the COVID-19 pandemic to overcome roadblocks in the access to cancer care. We propose that developing the domain of collaborative implementation research in national cancer control plans will be the key to consolidate patient-centred services with both an equity lens and a focus on integration of new technologies as all countries drive towards the 2030 goals of universal health coverage.

The COVID-19 (SARS-CoV-2 coronavirus disease) pandemic has hit cancer patients and services hard. Consistently around the globe, we have witnessed patients’ access to care being postponed and adjustments to treatments, delays in diagnosis as cancer screening programmes are suspended and key preventive services such as Human papillomavirus vaccination (HPV) vaccination being put on hold [1–3]. The global cancer community must unite and advocate for actions to mitigate and retain the cancer health gains made globally, illustrated by the fact that more countries than ever having established national cancer control plans and technical working groups to address the growing global cancer burden [4]. While projections predict at least a decade of impact on cancer outcomes in high-income settings [5, 6], recovery from COVID-19 will be especially hard in low- and middle-income countries (LMICs) where cancer systems will need to adapt and build resilience under persisting economic constraints [7].

In January 2021, Professor Gordon John McVie, champion of cancer research, medical oncologist, educator, patient advocate and an inspiration to many, died of complications due to COVID-19 and non-Hodgkin lymphoma, leaving a deep hole in the cancer community. Gordon’s footprint of driving public sector cancer research with a persistent focus on service delivery and impact on cancer outcomes, provides us with a blueprint to ‘building back better’. He would urge us to take forward a renewed focus on value-based change for improved cancer care, identifying the ‘silver linings’ lessons from the COVID-era [8] and integration of these learnings into global policy. The cancer community can do no better than look to build on Gordon’s legacy as i) a pioneer of patient-centred approaches and patient empowerment, ii) as a facilitator of networks with global reach for cross-discipline collaboration and iii) as an early adopter of innovation and technology to leverage the health systems’ focus required for us to maintain progress towards the 2030 sustainable development goals.

Gordon was known for never forgetting the person behind the patient. He worked for patients by working with patients and providing channels for the voice of people living with cancer, most recently through http://ecancerpatient.org. Key institutions which Gordon founded, led or collaborated with such as the European Organisation for Research and Treatment of Cancer, Clinical Trial Network, Cancer Research UK and European Institute of Oncology have embraced these principles, developing patient participation in the decision-making process as a gold standard in each phase of the cancer continuum (prevention, early diagnosis, treatment, palliative care and survivorship). In short, the empowered cancer patient is recognised as a driver of continued improvement in equity in access and quality of care illustrated by increased disease knowledge, adherence to medical treatment, satisfaction with the care and health outcomes, reducing direct and indirect costs for health systems [9–11].

Gordon went beyond sensibilisation alone and was devoted to designing and implementing strategies and infrastructures which enable a new culture in clinical practice based on patient empowerment as demonstrated by the European Union P-medicine Project, a technical platform able to manage clinical, psychological and social data to support implementation of personalised medicine in routine practice [12]. This innovative project shares tools to improve the patient–doctor relationship and joint decision-making processes. For example, the Interactive Empowerment Tool [13] is a web-based support to clinicians in improving their understanding of individual patient’s needs. Research on psychological health outcomes and integration of cultural and patient’s preferences in cancer services is however in its infancy in LMICs. An increasingly strong civil society movement is making the patient voice heard, particularly in the field of women cancers with breast and cervical cancers dominating the cancer statistics. As a global cancer community, we need to invest in these groups, to build their cancer research skills and ability to lead social science research but also to partner in the full suite of cancer research from basic science to implementation science. This need is stronger than ever before, noting the impact that the COVID-19 pandemic has on this valuable resource [14]. The field of survivorship especially, is underserved in developing countries. Once patients have left acute clinical care there is little follow-up, represented in the paucity of survival data beyond 12 months [15]. Gordon recognised this, taking steps to translate his own experience in the European setting towards capacity building and infrastructure in emerging economies. Specifically, Gordon supported the definition of pathways of care (POC) which allow for information transfer and a framework for implementation of change nationally. Evaluation of POC implementation projects in Australia [16] for example, has shown improved knowledge, awareness and use of POC as tools for monitoring service performance. Gordon was interested in translation of this methodology to Africa. Kenya is an example of a country taking the POC analytical approach forward, described in this recently published World Bank report [17]. Detailed patient interviews and focus group discussions were harnessed to better understand the direct and indirect costs families face, the difficult decisions and choices they need to make and the socioeconomic and psychological implications of having a family member afflicted by cancer.

Shared decision-making between patient and clinician, and systems that allow this to happen are a right not a privilege. Traditional approaches to medicine assume that patients and oncologists share the same set of values, expectations and preferences about cancer. Consequently, treatment decisions are exclusively focused on survival rate, are shaped on the ‘average patient’ and often override personal knowledge, values, needs, beliefs and emotions [3, 4]. Further, the dynamic nature of individual preferences which can adjust overtime related to environmental, social-economic, gender, cultural and individual conditions is sometimes neglected. Addressing these persistent false assumptions through a robust implementation research framework within cancer plans will be critical for building services sensitive to patient preferences and achieving optimal treatment outcomes at the individual level. Three major themes emerged in the Kenyan patient insights work [17] as current levers for improvement and further implementation research; firstly, the need for improving resilience of cancer patients as they embark on the road to recovery and second, the importance of cancer survivor groups to provide support. The third finding in Kenya was the potential impact of social safety nets to mitigate the impact on patients and households, underscoring the potential of patient views to leverage progress at systems level also. The importance for shaping the enabling environment for active community engagement in prevention, early detection and survivorship is well described in the World Health Organization global strategy to accelerate the elimination of cervical cancer [18], which will also require outreach to new communities of women living with HIV and integration of cancer services, building on existing services for HIV and maternal health.

‘*Why is it important?’* is a question that Gordon raised continually. Accruing evidence [19–21] has highlighted the importance to engage patients in each stage of the research process from defining the research agenda and allocation of the funding, engagement in the research teams, design and implementation of the clinical trials to dissemination of results and supporting the public understanding about the cancer research and its advancements. It is equally important to engage patients when translating research findings to routine clinical practice. Gordon was passionate about patient participation in cancer research, recognising the ability of the lived-experience to focus attention on sensitive cancer issues such as quality of care, social inequalities, and end-of-life care. Gordon encouraged patient insights towards improving the feasibility, appropriateness and consistency of cancer studies as well as enhance care delivery and patient outcomes [21]. As we look to recovery from the COVID-19 pandemic, it is critical that we do not assume that we are returning to business as usual. Changes in the ability of communities to adopt preventive risk reduction measures [22] and health seeking behaviours [23] reinforce the need for renewed research with patient involvement. Gordon was also passionate about bringing researchers of all disciplines and geographies together to fight cancer inequities. He recognised the key contribution of collaborative networks for integration of care to improve quality, efficiency and outcomes [24] and fought for their establishment in the UK [25], challenging them to do better and also establish new platforms for sharing knowledge and driving progress regionally and globally especially ecancer. Initiatives such as Project Extension for Community Healthcare Outcomes (ECHO) [26], Breast Health Global Initiative (BHGI) [27] and The International Collaboration for Research methods Development in Oncology (CReDO) [28] are examples of supporting cross-border and national networking towards research-based implementation which should include full patient involvement and from which we can learn for accelerating national efforts.

Key cancer learnings from the COVID-19 pandemic include the value of digital communication tools, a shift to self-care tools and the power of collaboration to accrue data and the evidence for decision-making on treatment. Gordon was a champion of harnessing proven effective health technologies [19, 20] that support full integration of patients in their care pathway. He underscored that transferring complex health concepts to patients and boosting active and responsible participation in clinical decision-making were the key to generating new clinical insights [29, 30]. Particularly, self-management tools have shown their worth during the COVID-19 pandemic, for example, use of the stool test for colorectal cancer screening and the HPV self-sample kits for cervical cancer screening have permitted continuation of critical early detection services despite disruptive pandemic control measures. Policy change to accelerate the uptake of these more convenient tools alongside issues such as decentralisation of cancer care should be a priority for improving equity of access to early detection services. Especially in low-and middle-income settings, critical scale-up of screening services to population-based approaches can benefit from leap-frogging older methodologies to high precision tests that are amenable to community-based care models [31]. Cancer civil society organisations, often early adopters of new technologies in their own work, have an important role to play in championing national infrastructure of cancer surveillance and research and in overcoming social inequalities and concerns about the use of technology in the clinical setting [30].

*‘Who pays?’* was another recurrent question from Gordon, whose aim was always for research to shape routine and sustainable cancer management services. Universal health coverage underpins the sustainable development goals and COVID-19, like no other crisis, has exposed health systems vulnerability, gaps in social protection and structural inequalities. While the importance of basic public health, and the resilience of a population in the face of any new virus or pandemic, lends ever greater urgency to the quest for universal health coverage [32], we must also acknowledge the new economic realities for many LMICs. The cancer community must rise to the challenge of ensuring all contributions to cancer prevention and control are optimised on the road to 2030. Gordon was foremost a clinician, but as a strong communicator and advocate he was the public face of the fight against cancer in the UK for many years and, when needed, a vocal critic of government cancer policy. The patient voice and cancer civil society groups in LMICs will need the support of the global cancer community in this time of crisis to maintain their organisations and drive local advocacy that counts, ‘channeling Gordon’ to focus on the pillars of equity of access and a health systems’ view of quality of care and outcomes assessment with affordability and financial protection at the core. A recent set of reports by the Cancer Alliance of South Africa [33] is an excellent example. Further, we should harness health systems’ research as an opener for career development and as mitigation against the potential for brain drain in the foreseeable harsh economic climate in LMICs [34].

## Conclusion

We conclude with a reminder that during the lead-up to the High-Level Meeting on noncommunicable diseases (NCDs) in 2011, the then Director General of the United Nations, Ban Ki-moon, warned of a tsunami of cancer and other NCDs describing it as a ‘public health emergency in slow motion’. The benefits of acting on NCDs by 2030 to health, economies and communities were the drivers for the global commitments made in the subsequent years – these motivations must not be forgotten. While evidence of patient and community insights has clearly contributed to improving cancer outcomes in the past decades, patient organisations and civil society groups still need to fight for what is often only a token engagement, a long way from being truly integrated into national cancer control plan processes and the research agenda in all countries. Patient and civil society networks in LMICs are having an impact, but their real concerns for survival in these challenging times call for us as a global cancer community to help them bridge the time to recovery from the COVID-19 pandemic and support their further development. Patient insights can and should play a key role in identifying gaps and new solution to aid prioritisation of activities in the response and recovery of the COVID pandemic in all settings but will be critical in LMICs to ensure that patient needs are at the centre of the response and recovery. As Gordon was well known to urge, we should trust that scientific, technological and clinical advancements will prevail, and we should trust innovative multi-stakeholder cancer research as the route to overcoming even the most challenging barriers. Investing in cancer research capacities and implementation research must underpin national cancer control plans in LMICs as we emerge from the COVID-19 pandemic. Inclusion of cancer advocates and patient representatives within these collaborative research networks will ensure the most urgent needs and gaps are prioritised as we work towards the 2030 health and sustainable development goals.

## Declaration of interests

Gabriella Prevattoni – no conflicts of interests to declare. Marianna Masiero – no conflicts of interests to declare. Christine Mugo-Sitati – no conflicts of interests to declare. Julie Torode, PhD – no conflicts of interests to declare.

## Funding declaration

The author(s) received no financial support for the research, authorship and/or publication of this article.

## Authors’ contributions

Contribution to design, writing and review: Gabriella Prevattoni, Marianna Masiero and Julie Torode.

Contribution to writing and review: Christine Mugo-Sitati.
